# Local-scale Partitioning of Functional and Phylogenetic Beta Diversity in a Tropical Tree Assemblage

**DOI:** 10.1038/srep12731

**Published:** 2015-08-03

**Authors:** Jie Yang, Nathan G. Swenson, Guocheng Zhang, Xiuqin Ci, Min Cao, Liqing Sha, Jie Li, J. W. Ferry Slik, Luxiang Lin

**Affiliations:** 1 Key Laboratory of Tropical Forest Ecology, Xishuangbanna Tropical Botanical Garden, Chinese Academy of Sciences, Kunming, China; 2 Department of Biology, University of Maryland, College Park, U.S.A; 3 University of Chinese Academy of Sciences, Beijing, China

## Abstract

The relative degree to which stochastic and deterministic processes underpin community assembly is a central problem in ecology. Quantifying local-scale phylogenetic and functional beta diversity may shed new light on this problem. We used species distribution, soil, trait and phylogenetic data to quantify whether environmental distance, geographic distance or their combination are the strongest predictors of phylogenetic and functional beta diversity on local scales in a 20-ha tropical seasonal rainforest dynamics plot in southwest China. The patterns of phylogenetic and functional beta diversity were generally consistent. The phylogenetic and functional dissimilarity between subplots (10 × 10 m, 20 × 20 m, 50 × 50 m and 100 × 100 m) was often higher than that expected by chance. The turnover of lineages and species function within habitats was generally slower than that across habitats. Partitioning the variation in phylogenetic and functional beta diversity showed that environmental distance was generally a better predictor of beta diversity than geographic distance thereby lending relatively more support for deterministic environmental filtering over stochastic processes. Overall, our results highlight that deterministic processes play a stronger role than stochastic processes in structuring community composition in this diverse assemblage of tropical trees.

Community ecologists frequently debate the relative degree to which deterministic and stochastic processes underpin community assembly. Neutral models of community assembly, for example, emphasize the importance of dispersal limitation and demographic stochasticity^1^. Deterministic models of community assembly, on the other hand, emphasize the importance of ecological and evolutionary differentiation between species and non-random responses of species to their biotic and abiotic environments^2^. Although the details of neutral and niche theory may differ among studies, relatively few studies have examined their relative importance^3^.

Beta diversity represents the compositional differentiation between species assemblages. Recent conceptual advances have been made using patterns of beta diversity to disentangle the relative importance of stochastic and deterministic processes during community assembly^4^,^5^,^6^,^7^. Beta diversity along spatial and environmental gradients, in particular, has been a focal point in the neutrality versus determinism debate. Specifically, community ecologists have often focused on quantifying the degree to which beta diversity can be explained by the geographic or environmental distance (or their combination) between two assemblages. In other words, ecologists often seek to partition the variation in species beta diversity into its geographic and environmental components^8^,^9^,^10^. A neutral model, that assumes dispersal limitation and no consequential ecological interactions with the environment, predicts that geographic distance will be the best predictor of species beta diversity. A deterministic model, that assumes the importance of ecological interactions with the environment, predicts that environmental distance will be the best predictor of species beta diversity. Thus, partitioning beta diversity into environmental and geographic components provides a useful tool for disentangling the relative importance of deterministic or neutral processes in community assembly^11^.

While beta diversity approaches have been widespread, they have largely focused on the species turnover or dissimilarity between assemblages^4^,^5^,^7^,^11^. However, such an approach assumes that all species independent, ignoring the functional and phylogenetic differences between them^12^,^13^. Thus it is impossible to identify whether the species turnover from one assemblage to the next are ecologically and evolutionarily similar or dissimilar, which can provide very different insights about the ecological and evolutionary processes that structure communities^14^,^15^. For example, one could simultaneously detect a high species turnover and a low turnover of functional strategies or lineages in diverse tropical forests due to the presence of many con-generic species and many functionally similar species. Thus, it is increasingly acknowledged that an analysis of only species is not sufficient to understanding the community assembly processes^13^.

Given the potential limitations of species beta diversity measures^13^,^15^, ecologists have begun to develop and implement measures of phylogenetic and functional beta diversity in microbial communities^16^, tropical tree communities^17^,^18^,^19^,^20^, and temperate plant communities^21^,^22^. Some of these studies have demonstrated instances where measures of species beta diversity do not unveil the underlying patterns. A clear early example of this comes from Fukami *et al.*^21^ who found that experimental plant communities may functionally converge through time while maintaining divergent species compositions indicating the importance of functional determinism. Further, recent work by Graham & Fine^15^, Swenson *et al.*^23^,^24^ and Siefert *et al.*^20^ has shown that phylogenetic and functional beta diversity analyses reveal signals of deterministic assembly processes that are not evident from patterns of species beta diversity alone.

Variation partitioning of regional-scale plant phylogenetic and functional beta diversity has successfully dissected the relative contribution of deterministic and stochastic processes^17^,^25^,^26^. However, local-scale investigations integrating both phylogenetic and functional turnover have been rare^24^,^27^,^28^, despite the large number of papers on phylogenetic and functional alpha diversity in plant communities^29^,^30^,^31^.

Here we aim to provide a detailed analysis of the phylogenetic and functional beta diversity on local scales for tropical trees. Specifically, we utilized a large forest dynamics plot in tropical China. Species level functional trait data were collected and a molecular community phylogeny was generated for over 400 co-occurring species in this plot. The distribution, environment, trait and phylogenetic data were used to quantify whether environmental (soil nutrients) distance, geographic distance or their combination are stronger predictors of phylogenetic and functional beta diversity in tropical trees. Because many phylogenetic and functional patterns in tropical trees have been shown to be scale dependent^30^,^32^, all analyses were replicated across multiple spatial scales. Further, we compared the phylogenetic and functional beta diversity within and between habitats to determine whether a coarser scale categorical perspective of the environment can provide results comparable to those generated from fine scale continuous representations of the environment.

## Results

### Phylogenetic and functional beta diversity

**Figure 1:**
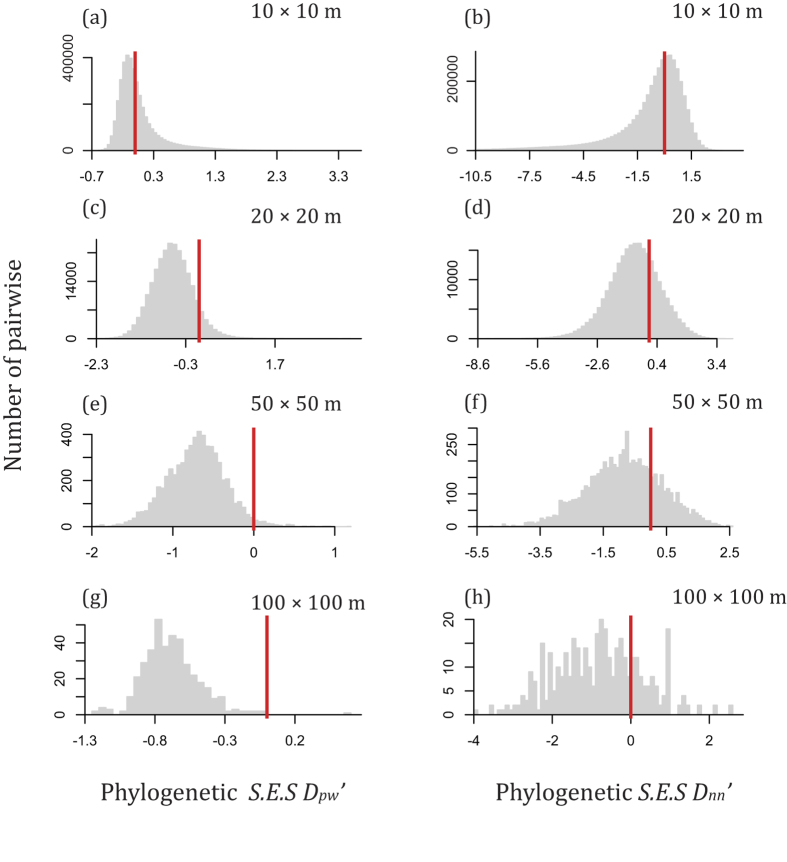
The distribution of standard effective size for abundance-weighted mean pairwise phylogenetic distance (*S.E.S. D*_*pw*_’) and standard effective size for abundance-weighted mean nearest taxon phylogenetic distance (*S.E.S. D*_*nn*_’) across scales. Bars to the left of the red zero line indicates phylogenetic turnover is faster than expected. The proportions of S.E.S values below zero were as follows: (**a**) 57.16%, (**b**) 57.35%, (**c**) 91.18%, (**d**) 71.96%, (**e**) 97.80%, (**f**) 75.81%, (**g**) 99.75%, (**h**) 76%.

**Figure 2:**
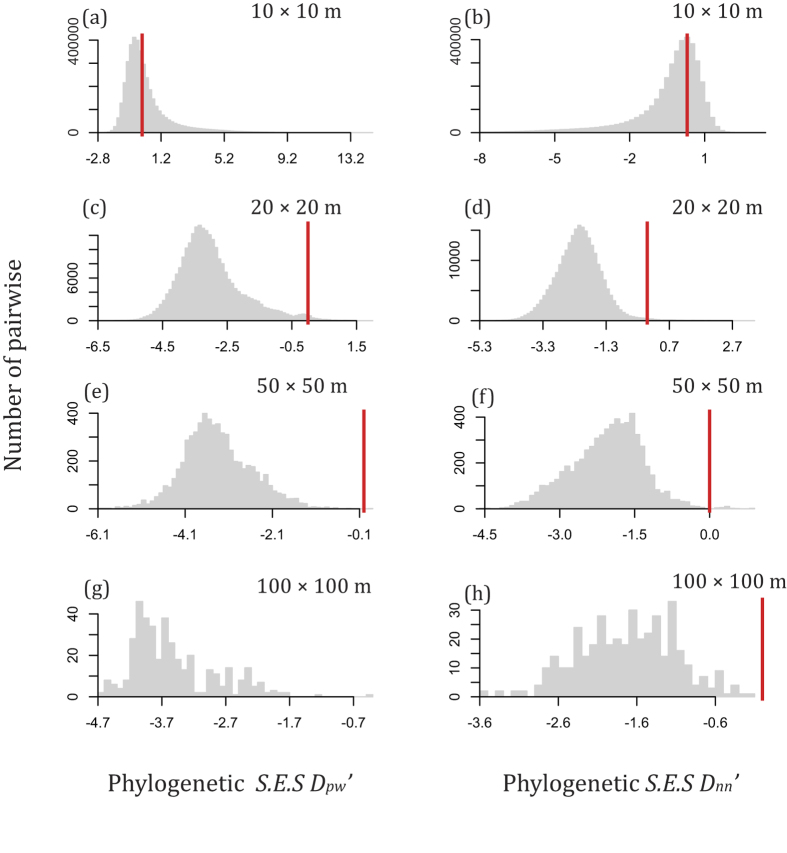
The distribution of standard effective size for abundance-weighted mean pairwise functional distance (*S.E.S. D*_*pw*_’) and standard effective size for abundance-weighted mean nearest taxon functional distance (*S.E.S. D*_*nn*_’) across scales. Bars to the left of the red zero line indicates functional turnover is faster than expected. The proportions of S.E.S values below zero were as follows: (**a**) 58.35%, (**b**) 61.26%, (**c**) 94.7%, (**d**) 94.84%, (**e**) 99.14%, (**f**) 95.91%, (**g**) 100%, (**h**) 98.25%.

In general, the values of *S.E.S. D*_*pw*_ and *S.E.S. D*_*nn*_ deviated from zero across spatial scales (Figs 1 and 2). In most cases, the values of *S.E.S. D*_*pw*_ and *S.E.S. D*_*nn*_ were less than zero (Figs 1 and 2), indicating that the phylogenetic and functional dissimilarity between subplots was often higher than expected. However, there were also some cases of positive *S.E.S. D*_*pw*_ and *S.E.S. D*_*nn*_, indicating that lower phylogenetic and functional turnover than expected also occurred. High phylogenetic and functional turnover between pairs of subplots was observed using indices based on species abundances and based on species occurrences (Figs S3 and S4). These patterns of non-random phylogenetic and functional beta diversity are consistent with habitat specialization being an important process in community assembly. We also found that the proportion of negative *S.E.S. D*_*pw*_values was generally higher than that of negative *S.E.S. D*_*nn*_values across spatial scales (Figs 1 and 2).

### Phylogenetic and functional beta diversity across habitats

**Figure 3:**
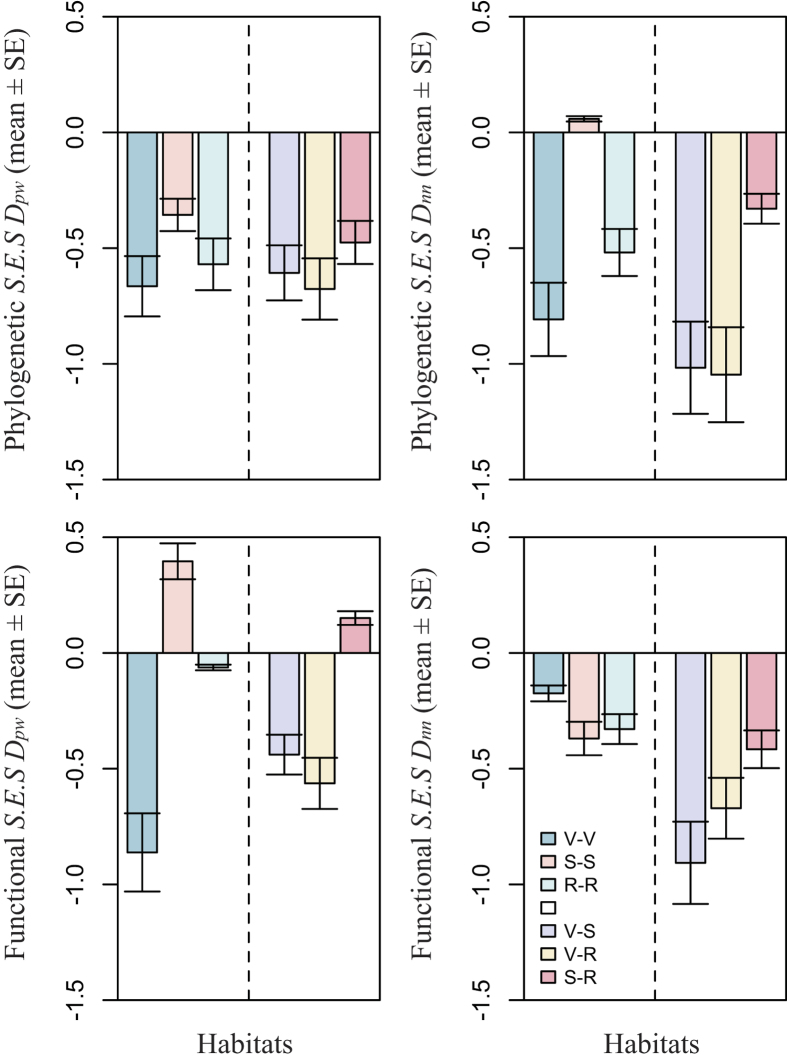
Bar charts illustrating phylogenetic and functional dissimilarity between subplot (20 m × 20 m) pairs both within habitats and across habitats. V: Valley; S: Slope; R: Ridge. The same letters represent phylogenetic and functional dissimilarity within habitats (i.e. V-V, S-S, R-R) and the different letters represent that across habitats (i.e. V-S, V-R, S-R).

In general, phylogenetic and functional turnover was higher than expected by chance both within or across habitat types as indicated by predominantly negative *S.E.S. D*_*pw*_ and *S.E.S. D*_*nn*_ values (Fig. 3). However, we found that the phylogenetic and functional turnover within habitat types was significantly lower than that between habitat types (Table S5).

### Relationships between phylogenetic and functional dissimilarity and geographic and environmental distance

The phylogenetic and functional dissimilarity between subplots was significantly correlated to geographic and environmental distance, except at the scale of 10 × 10 m (Tables 1, 2, S3 and S4). The explanatory power of geographic and environmental distance increased with spatial scales. The multiple regressions on distance matrices (MRM) results demonstrated that only a small proportion of variation in phylogenetic or functional dissimilarity using both *D*_*pw*_ and *D*_*nn*_ could be explained by geographic distance together with environmental distance on the finest scale (10 × 10 m), but about 40% of the variation in phylogenetic or functional turnover (*D*_*nn*_) at the scale of 100 × 100 m can be explained by combination of environmental and geographic distance (Tables 1 and 2). Environmental distance explained much more variation than geographic distance using both the *D*_*pw*_ and *D*_*nn*_ metrics (Tables 1 and 2). Lastly, the amount of variation in *D*_*pw*_ explained by environmental distance was less than that explained for *D*_*nn*_ by environmental distance (Tables 1 and 2).

## Discussion

The present study focused on quantifying the phylogenetic and functional beta diversity and partitioning their variation along environmental and spatial gradients at multiple spatial scales in a Chinese tropical forest. We found that the amount of variation in phylogenetic and functional beta diversity explained by environmental distance was consistently greater than that explained by geographic distance. Our results provided strong support for a deterministic model underlying the beta diversity of trees in our forest.

The first goal of this study was to quantify the degree to which the observed phylogenetic or functional beta diversity differed from that expected given the observed species beta diversity. This part of the study was accomplished by performing null model analyses across four spatial scales. A neutral model would predict that the observed phylogenetic and functional beta diversity should not deviate from that expected given the observed species beta diversity^15^,^23^. Our results show that the observed phylogenetic and functional beta diversity was generally greater than that expected given the species turnover as indicated by the negative *S.E.S. D*_*pw*_ and *S.E.S. D*_*nn*_ in most cases (Figs 1 and 2). This result indicates that niche-based deterministic processes are relatively more important in structuring the tree community studied than neutral processes^15^,^20^,^24^,^33^.

The second goal of this study was to partition the amount of variation in phylogenetic and functional beta diversity with respect to environmental and geographic distance. The MRM analysis showed the phylogenetic and functional dissimilarity between subplots were significantly correlated with both geographic and environmental distance except the scale of 10 × 10 m (Tables 1 and 2). The pure spatial component as the explanatory variable is often interpreted as evidence of dispersal limitation and the potential importance of neutrality^17^,^26^. The explanatory power of geographic distance can be explained by not only dispersal limitation but also the spatially structured environmental variables^34^,^35^,^36^ not included in our analyses. However, the explanatory power of environmental distance was generally greater than geographic distance across spatial scales (Tables 1 and 2). This is consistent with the rapid turnover in lineages and functions through space in our forest plot which has rugged terrain with rapid changes in topography and the abiotic environment over short spatial distances (Figs 1 and 2). Thus, we can conclude that environmental filtering plays a more important role in driving the phylogenetic and functional turnover in our forest than neutral processes. Our results also support previous findings that phylogenetic or functional turnover is significantly related to environmental gradients from local to regional scales^26^.

On very fine spatial scales 100 m^2^ little variation in phylogenetic or functional beta diversity could be predicted by the geographic distance or environmental dissimilarity based upon the soil variables measured. This result is most likely driven by one to three possible, not necessarily mutually exclusive, possibilities. First, random dispersal on this local scale and a lack of strong interactions between species and their biotic and abiotic environments could result in a spatially heterogenous (i.e. non-autocorrelated) turnover. Second, unmeasured environmental variables that are not spatially autocorrelated (e.g. vertical light gradients or fine scale soil nutrient gradients) may be important on this local scale. Third, biotic interactions promote the consistent dissimilarity between species within subplots (i.e. high phylogenetic or functional alpha diversity) in similar environments thereby reducing the overall phylogenetic or functional turnover on very local scales. It is most likely that all three of these possibilities are important in the forest plot and future more detailed analyses on very fine spatial scales are required.

Habitat specialization is often thought to influence taxonomic, phylogenetic and functional turnover in tropical forests^5^,^17^,^24^. Given this expectation we further examined the phylogenetic and functional beta diversity within and across the three different broad categorical habitat types in the plot. If these habitats filter lineages and functions, we would expect that the phylogenetic and functional beta diversity within habitats would be lower than expected by positive *S.E.S. D*_*pw*_ and *S.E.S. D*_*nn*_. Further, we might expect this to result in faster phylogenetic and functional turnover than expected across habitats as indicated by negative *S.E.S. D*_*pw*_ and *S.E.S. D*_*nn*_. However, we found *S.E.S. D*_*pw*_ and *S.E.S. D*_*nn*_ were generally negative whether within or across habitats indicating a higher than expected turnover (Fig. 3). Taken together with the evidence of how variance in phylogenetic and functional beta diversity is partitioned along continuous soil nutrient axes in this forest, it suggests that local scale environmental heterogeneity within habitats plays a predominant role in structuring the phylogenetic and functional turnover in this forest.

Our phylogenetic and functional beta diversity results were generally consistent, though there were slight differences that are likely explained by the fact that most functional traits had weak, albeit significant, phylogenetic signal^37^. As many researchers have argued, phylogenetic similarity is not always a good predictor of ecological similarity^38^, and thus only using phylogenetic information may be misleading when coexisting species simultaneously converge and diverge in function^13^. Here we calculated the phylogenetic and functional dissimilarity by using the algorithms of pairwise distance (*D*_*pw*_) and nearest neighbor distances (*D*_*nn*_). The *D*_*pw*_ and *D*_*nn*_ are ‘basal’ and ‘terminal’ metrics of phylogenetic or functional beta diversity^18^. Although there were subtle differences between *D*_*pw*_ and *D*_*nn*_, our results based on these two metrics were generally consistent (Figs 1 and 2). However, *S.E.S. D*_*pw*_seems to reflect stronger phylogenetic and functional dissimilarity across four spatial scales than did *D*_*nn*_ (Figs 1 and 2). The generally negative *S.E.S D*_*pw*_ and *S.E.S D*_*nn*_ across spatial scales indicate that phylogenetic and functional turnover was faster than that expected by chance given the species turnover in both of distantly related species (basal turnover) and species within terminal clades (terminal turnover). However, less of the variation in the *S.E.S. D*_*pw*_ values could be explained by environmental distance, geographic distance, and their combination than *D*_*nn*_ (Tables 1 and 2). This result was also consistent with the studies of phylogenetic dissimilarity of 96 tree communities in India^18^ and in the Gutianshan forest dynamics plot in China^39^.

## Methods

### Study site

This study was conducted in the 20-ha Xishuangbanna forest dynamics plot (FDP) located in the Yunnan Province, southwest China (21°36′N, 101°34′E) (Fig. S1). This forest is characterized as a seasonal tropical rainforest and dominated by large individuals of *Parashorea chinensis* (Dipterocarpaceae). There are 111,177 stems and 468 species of trees in this plot. The climate is strongly seasonal with distinct alterations between the dry season (from May to October) and the wet season (from November to April). The annual mean precipitation is 1493 mm, of which 1256 mm (84%) occurs in the wet season^37^. Elevation within the FDP ranges from 708.2 m to 869.1 m. The Xishuangbanna FDP was established in 2007 where all freestanding woody stems ≥1 cm diameter at 130 cm from the ground DBH (Diameter at Breast Height) were measured, mapped, tagged and identified to species between November 2006 and April 2007. A detailed description of the climate, geology and flora of Xishuangbanna can be found in Cao *et al.*^40^.

### Functional traits

Trait data for the tree species in the plot were collected using standardized protocols^41^ with the exception of leaf chlorophyll content and stem specific resistance. Leaf area, specific leaf area (SLA), leaf thickness, leaf dry matter content (LDMC) and leaf chlorophyll content are regarded as key traits related to resource allocation strategies^41^,^42^. Maximum tree height is directly related to the adult light niche^43^. Wood density represents a tradeoff between volumetric growth and mechanical vulnerability on the tissue and organismal scale and it is often the trait most closely related to hydrological niche and demographic rates^44^. Stem specific resistance has been shown to be strongly correlated with wood density and was therefore used as a substitute^45^. Seed mass represents a trade-off between producing many small seeds per unit energy versus producing a few large seeds per unit energy^46^. In total, we measured eight functional traits that are believed to represent fundamental functional trade-offs in leaves, wood and seeds among tree species^47^. We used the mean values of traits for each tree species in this study. Detailed information regarding the collection and measurement of functional traits is provided in Yang *et al.*^37^.

### Soil nutrients

Soil samples were collected and analyzed for the Xishuangbanna FDP following the standardized protocols described in John *et al.*^48^. Specifically, soil was sampled using a regular grid of 30 × 30 m in the 20-ha plot. The 252 grid intersections were basal collection points, together with each base point, three additional sampling points were located at random combination of 2 and 5 m, 2 and 15 m or 5 and 15 m along a random compass bearing away from the associated base point. At each sample point, we removed the litter and humus layer, then collected 500 g topsoil from depth of 0–10 cm. A total of 756 soil samples were taken. Fresh soil samples were placed into pre-labeled plastic bags and shipped to the Biogeochemistry Laboratory at the Xishuangbanna Tropical Botanical Garden. In the laboratory, one sub-sample was used for measuring pH values as immediately as possible using a potentiometer in fresh soil after water extraction (soil : water was 1 : 2.5). The other sub-samples were air-dried, smashed, sieved using 1 mm and 0.15 mm mesh and stored in plastic bags for soil bulk density, total C, total N, total P, total K, available N, extractable P and extractable K analysis. Soil sample data were subjected to variogram modeling, which was then used in block kriging to estimate mean soil nutrient concentrations in each subplot^48^,^49^. Finally, we obtained the spatial maps of average soil water content and 9 soil nutrients (pH, total N, total P, total K, available N, extractable P, extractable K, total C, bulk density) in the plot. Using the soil maps we characterized the environment of each subplot at each spatial scale. Additional detailed information regarding the measurement of soil variables can be found in Hu^49^.

### Phylogenetic tree reconstruction

We reconstructed a community phylogeny representing 428 of the species in the Xishuangbanna FDP (Fig. S2). A DNA supermatrix was generated from three chloroplast sequence regions – *rbcL*, *matK*, *trnH*-*psbA* and nuclear ribosomal internal transcribed spacer (ITS)^50^. The DNA supermatrix was then analyzed using RAxML via the CIPRES supercomputer cluster to infer a maximum likelihood (ML) phylogeny using the APG III phylogenetic tree as a constraint or guide tree as described in Kress *et al.*^50^. Finally, we implemented non-parametric rate smoothing (NPRS) in the software r8s^51^. Detailed information regarding the phylogenetic tree reconstruction is provided in Yang *et al.*^37^.

### Phylogenetic and functional beta diversity

We sequentially divided the 20-ha plot into 2000 10 × 10 m, 500 20 × 20 m, 80 50 × 50 m and 20 100 × 100 m subplots. The beta diversity analyses were performed between subplots using the above multiple spatial scales. To eliminate trait redundancy, we performed a principle components analysis (PCA) on the functional trait data (Table S1). We utilized the first three principle components, that explain over 70% of the variation in the trait data, to construct a Euclidean trait distance matrix. An Unweighted Pair Group Method with Arithmetic Mean (UPGMA) hierarchical clustering was then applied to this matrix to produce a trait dendrogram^52^. This dendrogram represented the trait similarity between taxa^23^.

Based on the molecular phylogenetic tree and trait dendrogram, we calculated the phylogenetic and functional dissimilarity between each pair of subplots using the mean pairwise phylogenetic or trait distance (*D*_*pw*_) and the mean nearest neighbor phylogenetic or trait distance (*D*_*nn*_)^53^,^54^. We also calculated abundance-weighted mean pairwise phylogenetic or trait distance (*D*_*pw*_’) and abundance-weighted mean nearest neighbor phylogenetic or trait distance (*D*_*nn*_’). *D*_*pw*_, *D*_*nn*_, *D*_*pw*_’ and *D*_*nn*_’ are calculated as follows:


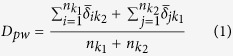



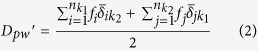



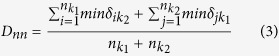



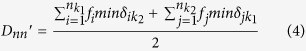


Where 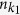
represents the number of species in community *k*_*1*_; 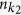 represents the number of species in community *k*_*2*_; 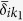 is the mean pairwise phylogenetic or trait distance between species *i* in community *k*_*1*_ to all species in community *k*_*2*_ and 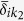 is the mean pairwise phylogenetic or trait distance between species *j* in community *k*_*2*_ to all species in community *k*_*1*_; where min 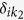
is the nearest neighbor phylogenetic or trait distance between species *i* in community *k*_*1*_ to all species in community *k*_*2*_ and min 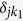 is the nearest neighbor phylogenetic or trait distance between species *j* in community *k*_*2*_ to all species in community *k*_*1*_, where *f*_*i*_ and *f*_*j*_ are the relative abundance of species *i* in community *k*_*1*_ and species *j* in community *k*_*2*_*. D*_*pw*_ and *D*_*pw*_’ are ‘basal’ metrics of phylogenetic or trait beta diversity, while *D*_*nn*_ and *D*_*nn*_’ are ‘terminal’ metric of phylogenetic or trait beta diversity^18^.

Although there are many methods for calculating phylogenetic and functional beta diversity^33^, the advantage of *D*_*pw*_ and *D*_*nn*_ is that they represent the two main mathematically independent classes of phylogenetic and functional dissimilarity metrics while avoiding the redundancy of calculating other nearly identical metrics^17^,^18^.

We addressed whether the observed phylogenetic or functional dissimilarity deviated from a random expectation by comparing the observed values with 999 null values generated by a null model. The null model randomly shuffled the names of species across the tips of the phylogenetic tree or trait dendrogram 999 times. This approach randomized the phylogenetic relatedness or trait similarity of species to one another, while maintained the observed community data matrix. Thus, species occupancy frequencies, abundances and spatial distributions (e.g. any patterns of intraspecific aggregation or dispersal limitation) were fixed in each randomization.

A standardized effect size of *D*_*pw*_ (*S.E.S. D*_*pw*_) and the standardized effect size of *D*_*nn*_ (*S.E.S. D*_*nn*_) were quantified using the null distribution^18^,^20^,^24^,^39^. Specifically, the mean of the null distribution was subtracted from the observed value and divided by the standard deviation of the null distribution. This value was then multiplied by negative one to stick with convention. Thus, negative *S.E.S.D*_*pw*_ or *S.E.S.D*_*nn*_ values indicated a higher observed *D*_*pw*_ or *D*_*nn*_ than expected and positive *S.E.S.D*_*pw*_ or *S.E.S.D*_*nn*_ values indicated a lower observed *D*_*pw*_ or *D*_*nn*_ than expected. We calculated the *S.E.S.D*_*pw*_ and *S.E.S.D*_*nn*_ for each pair of subplots at each spatial scale.

Lastly, we were also interested in whether the phylogenetic and functional beta diversity between subplots in the same habitat type was lower than that between habitat types. To quantify this, the 20-ha plot was divided into three habitat types using the topographic variables including slope, elevation and convexity (Table S2). The three habitat types, valley, slope and ridge, were assigned to each 20 × 20 m subplot in the FDP. The spatial distribution of three habitat types is given in Figure S1. We calculated phylogenetic and functional *S.E.S. D*_*pw*_ and *S.E.S D*_*nn*_, for each pair of subplots within the same habitats and that across the different habitats, and then use Student’s t test to compare the means of *S.E.S. D*_*pw*_ or *S.E.S D*_*nn*_ between within habitats and across habitats.

### Variation partitioning of phylogeneitc and functional beta diversity

We utilized MRM^55^,^56^ to relate phylogenetic or functional dissimilarity (*D*_*pw*_ or *D*_*nn*_) with geographic distance and environmental distance. MRM is similar to a partial Mantel’s test and can be used to examine the correlation between the dependent distance matrix and the independent explanatory distance matrices. Next we used variation partitioning to determine the explanatory power of independent effects and joint effects of the explanatory factors^8^. We constructed the geographic distance matrix by calculating the geographic distance between each pair of subplots using the coordinates in the center of each subplot. A PCA was conducted on all soil nutrient variables and an environmental distance matrix was then constructed by the Euclidean distance between each pair of subplots based on the first three principle components. The above analyses were performed using the R packages “ecodist”, “vegan” and “picante”^57^,^58^,^59^,^60^.

## Additional Information

**How to cite this article**: Yang, J. *et al.* Local-scale Partitioning of Functional and Phylogenetic Beta Diversity in a Tropical Tree Assemblage. *Sci. Rep.* **5**, 12731; doi: 10.1038/srep12731 (2015).

## Supplementary Material

Supplementary Information

## Figures and Tables

**Table 1:** The variation of phylogenetic dissimilarity explained by geographic and environmental distances across scales using multiple regressions on distance matrices.

Scale (m^2^)	Response distance matrix	**Explanatory distance matrices**		
		**Combination of geographic and environmental distance**	**Geographic distance**	**Environmental distance**
10 × 10	*D*_*pw*_’	0.004*	0.003	0.001
	*D*_*nn*_’	0.009*	0.009	0.000
20 × 20	*D*_*pw*_’	0.139***	0.005***	0.139***
	*D*_*nn*_’	0.207***	0.027***	0.197***
50 × 50	*D*_*pw*_’	0.199***	0.012***	0.199***
	*D*_*nn*_’	0.279***	0.072***	0.246***
100 × 100	*D*_*pw*_’	0.244***	0.018***	0.235***
	*D*_*nn*_’	0.410***	0.177***	0.385***

***P < 0.001, **P < 0.01, *P < 0.05.

“Combination of geographic and environmental distance” represents the whole variation explained by the geographic and environmental components; “Geographic distance” represents the pure variation explained by the spatial component; “Environmental distance” represents the pure variation explained by the soil nutrients component.

**Table 2:** The variation of functional dissimilarity explained by geographic and environmental distances across scales using multiple regressions on distance matrices.

Scale (m^2^)	Response distance matrix	**Explanatory distance matrices**		
		**Combination of geographic and environmental distance**	**Geographic distance**	**Environmental distance**
10 × 10	*D*_*pw*_’	0.006	0.002	0.003
	*D*_*nn*_’	0.012*	0.011	0.001
20 × 20	*D*_*pw*_’	0.172***	0.006***	0.166***
	*D*_*nn*_’	0.224***	0.040***	0.184***
50 × 50	*D*_*pw*_’	0.201***	0.007***	0.201***
	*D*_*nn*_’	0.271***	0.074***	0.236***
100 × 100	*D*_*pw*_’	0.202***	0.026***	0.200***
	*D*_*nn*_’	0.401***	0.121***	0.396***

***P < 0.001, **P < 0.01, *P < 0.05.

“Combination of geographic and environmental distance” represents the whole variation explained by the spatial and environmental components; “Geographic distance” represents the pure variation explained by the spatial component; “Environmental distance” represents the pure variation explained by the environmental component.
