# Supplementary material for: Local-scale Partitioning of Functional and Phylogenetic Beta Diversity in a Tropical Tree Assemblage
Source: Sci Rep. 2015 Aug 3;5:12731. doi: 10.1038/srep12731 (PMC4522671; doi:10.1038/srep12731)
Supplement: Supplementary Information [file srep12731-s1.pdf]

Local-scale Partitioning of Functional and Phylogenetic Beta Diversity in a Tropical Tree Assemblage

Jie Yang[1], Nathan G. Swenson[2], Guocheng Zhang[1,3], Xiuqin Ci[1,3], Min Cao[1], Liqing Sha[1], Jie Li[1], J. W. Ferry Slik[1], Luxiang Lin[1]*

[1]Key Laboratory of Tropical Forest Ecology, Xishuangbanna Tropical Botanical Garden, Chinese Academy of Sciences, Kunming, China, [2]Department of Plant Biology, Michigan State University, East Lansing, Michigan, U.S.A., [3]University of Chinese Academy of Sciences, Beijing, China

* Correspondence and requests for materials should be addressed to L.L. (linluxa@xtbg.ac.cn)

Figure legends

Figure S1 Geographical location and the spatial distribution of the three habitat types in 20-ha Xishuangbanna forest dynamics plot. Colors represent different habitat types at the spatial scale of 20 × 20 m. The map was generated using DIVA-GIS 7.5.

Figure S2 The phylogenetic tree constructed from DNA barcodes of the 428 species in the 20-ha Xishuangbanna forest dynamics plot.

Figure S3 The distribution of standard effective size for non-abundance-weighted mean pairwise phylogenetic distance (*S.E.S.* D_{pw}) and standard effective size for non-abundance-weighted mean nearest taxon phylogenetic distance (*S.E.S.* D_{nn}) across scales. Bars to the left of the red zero line indicates phylogenetic turnover is faster than expected. The proportions of S.E.S values below zero were as follows: (a) 65.16%, (b) 77.35%, (c) 51.28%, (d) 81.76%, (e) 47.80%, (f) 85.81%, (g) 69.75%, (h) 94.56%.

Figure S4 The distribution of standard effective size for non-abundance-weighted mean pairwise functional distance (*S.E.S.* D_{pw}) and standard effective size for non-abundance-weighted mean nearest taxon functional distance (*S.E.S.* D_{nn}) across scales. Bars to the left of the red zero line indicates functional turnover is faster than expected. The proportions of S.E.S values below zero were as follows: (a) 56.35%, (b) 71.26%, (c) 95.6%, (d)

95.48%, (e) 98.02%, (f) 95.91%, (g) 99.6%, (h) 90.25%.

Table S1 The loadings of eight continuous functional traits on the first three principal components for 428 tree species in the 20-ha Xishuangbanna forest dynamics plot.

Functional traits	PCA1	PCA2	PCA3
LDMC	-0.534	-0.008	-0.376
Leaf area	0.302	0.499	0.278
Leaf chlorophyll content	-0.165	0.362	0.005
Leaf thickness	0.091	0.696	-0.113
Maximum height	-0.273	0.112	0.553
Seed mass	-0.336	0.014	0.544
SLA	0.522	-0.325	0.303
Stem specific resistance	-0.358	-0.130	0.274
% explained	49.961	12.264	10.514

SLA, specific leaf area; LDMC, leaf dry mass content.

Table S2 Classification criteria for the three topographical habitat types for all 20 × 20 m quadrats in the 20-ha Xishuangbanna forest dynamics plot.

Habitats	Valley	Slope	Ridge
Elevation (m)	< 779.8	all	all
Slope (degree)	all	≥ 27.14	< 27.14
Convexity (degree)	< 0	≥ 0	all

27.14 and 779.8 are median slope and median elevation respectively for all 20 × 20 m quadrats within the plot.

Table S3 The variation of phylogenetic dissimilarity explained by geographic and environmental distances across scales using multiple regressions on distance matrices based on non-abundance-weighted metrics.

Scale (m^2)	Response distance matrix	Explanatory distance matrices		
		Combination of geographic and environmental distance	Geographic distance	Environmental distance
10 × 10	D_{pw}	0.096***	0.002***	0.096***
	D_{nn}	0.089*	0.010	0.087
20 × 20	D_{pw}	0.004***	0.004	0.000***
	D_{nn}	0.207***	0.027***	0.197***
50 × 50	D_{pw}	0.174***	0.007	0.173***
	D_{nn}	0.144***	0.064***	0.114***
100 × 100	D_{pw}	0.196***	0.018	0.235***
	D_{nn}	0.325***	0.150***	0.300***

*** P < 0.001, ** P < 0.01, * P < 0.05

Table S4 The variation of functional dissimilarity explained by geographic and environmental distances across scales using multiple regressions on distance matrices based on non-abundance-weighted metrics.

Scale (m^2)	Response distance matrix	Explanatory distance matrices		
		Combination of geographic and environmental distance	Geographic distance	Environmental distance
10 × 10	D_{pw}	0.004	0.000	0.004
	D_{nn}	0.036*	0.008	0.033
20 × 20	D_{pw}	0.016***	0.008***	0.007***
	D_{nn}	0.020***	0.019***	0.000***
50 × 50	D_{pw}	0.058***	0.007***	0.058***
	D_{nn}	0.142***	0.068***	0.108***
100 × 100	D_{pw}	0.000***	0.000***	0.000***
	D_{nn}	0.242***	0.080***	0.236***

*** P < 0.001, ** P < 0.01, * P < 0.05

Table S5 One-tailed student's t test for whether the mean phylogenetic and functional dissimilarity within habitats was lower than that across habitats.

Habitats	Phylogenetic *S.E.S* D_{pw}	Phylogenetic *S.E.S* D_{nn}	Functional *S.E.S* D_{pw}	Functional *S.E.S* D_{nn}
	P value	*P* value	*P* value	*P* value
VV-VS	0.530	**0.000**	**0.023**	**0.001**
VV-VR	**0.001**	**0.001**	**0.001**	**0.001**
VV-SR	**0.001**	**0.000**	**0.001**	**0.001**
SS-VS	**0.005**	**0.000**	**0.001**	**0.001**
SS-VR	**0.001**	**0.000**	**0.001**	**0.001**
SS-SR	**0.001**	**0.000**	**0.001**	**0.001**
RR-VS	**0.001**	**0.001**	**0.001**	**0.001**
RR-VR	**0.001**	**0.001**	**0.001**	**0.001**
RR-SR	0.236	0.068	0.673	0.041

V: Valley; S: Slope; R: Ridge. The same letters represent phylogenetic and functional dissimilarity within habitats (i.e. VV, SS, RR) and the different letters represent that across habitats (i.e. VS, VR, SR). $P < 0.025$ is shown in bold.

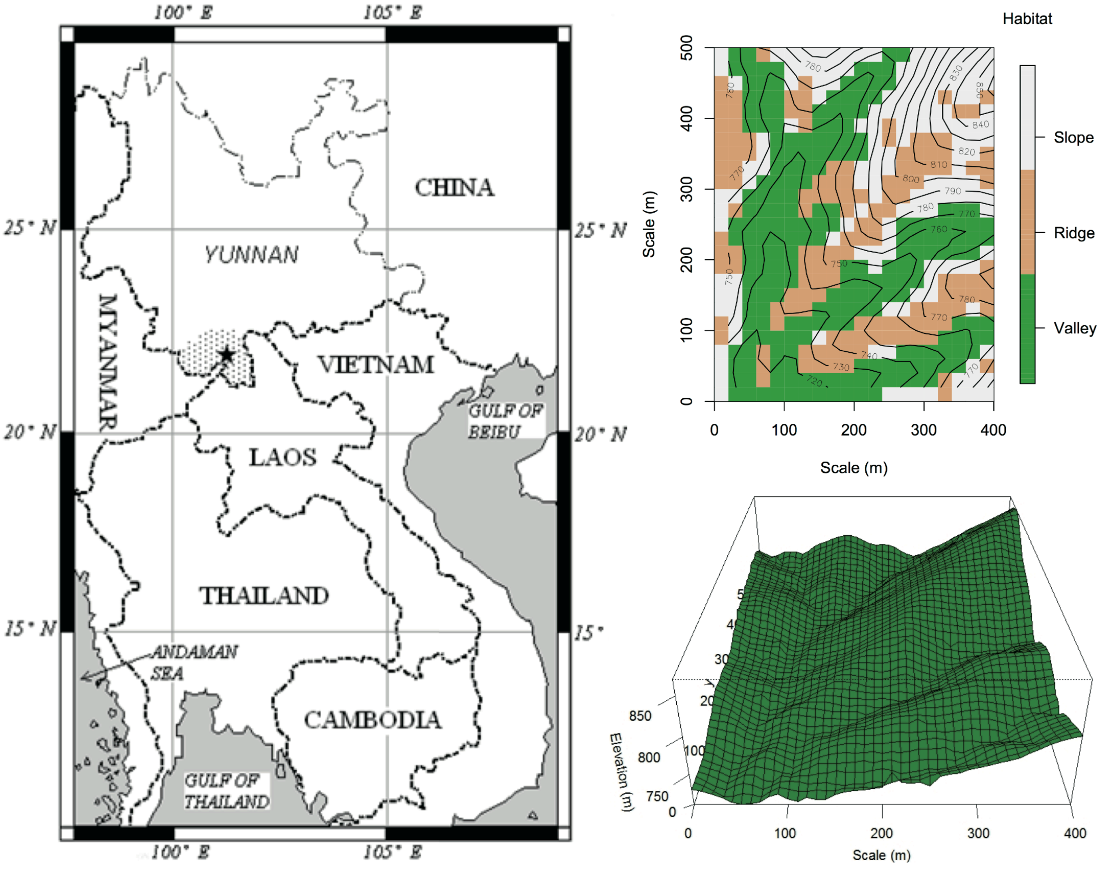

Figure S1 Geographical location and the spatial distribution of the three habitat types in 20-ha Xishuangbanna forest dynamics plot. Colors represent different habitat types at the spatial scale of 20 × 20 m. The map was generated using DIVA-GIS 7.5.

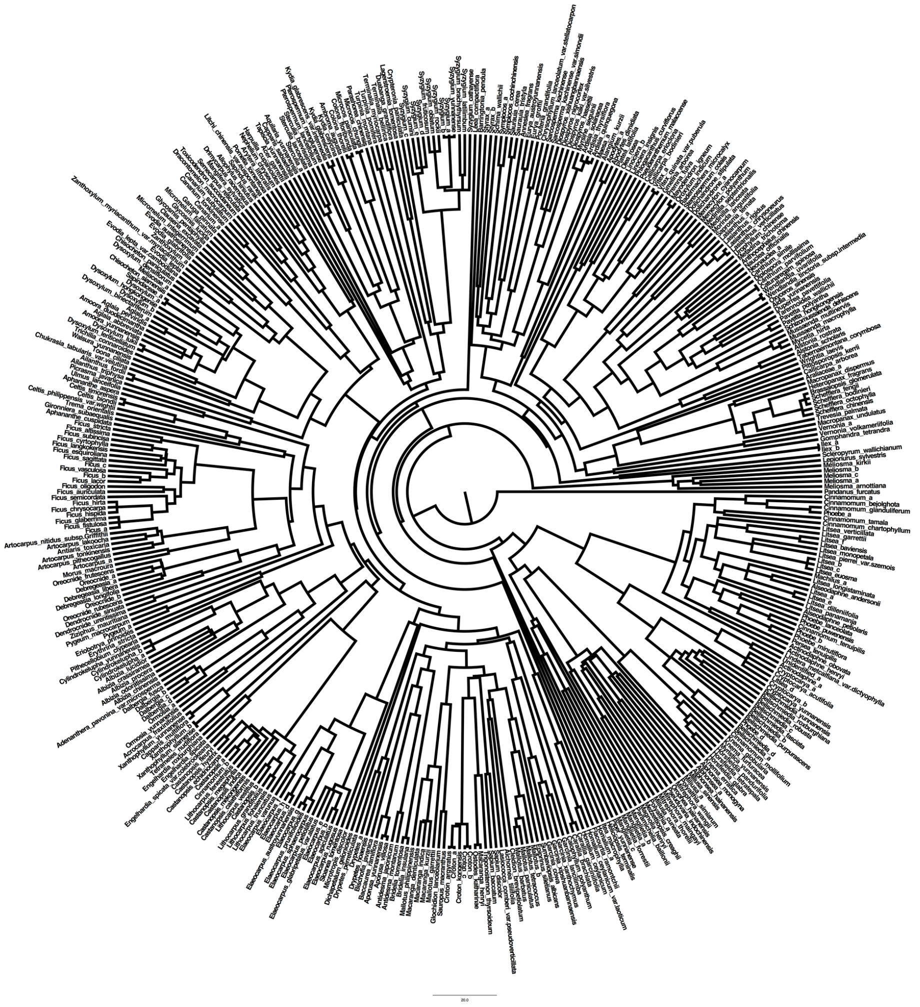

Figure S2 The phylogenetic tree constructed from DNA barcodes of the 428 species in the 20-ha Xishuangbanna forest dynamics plot.

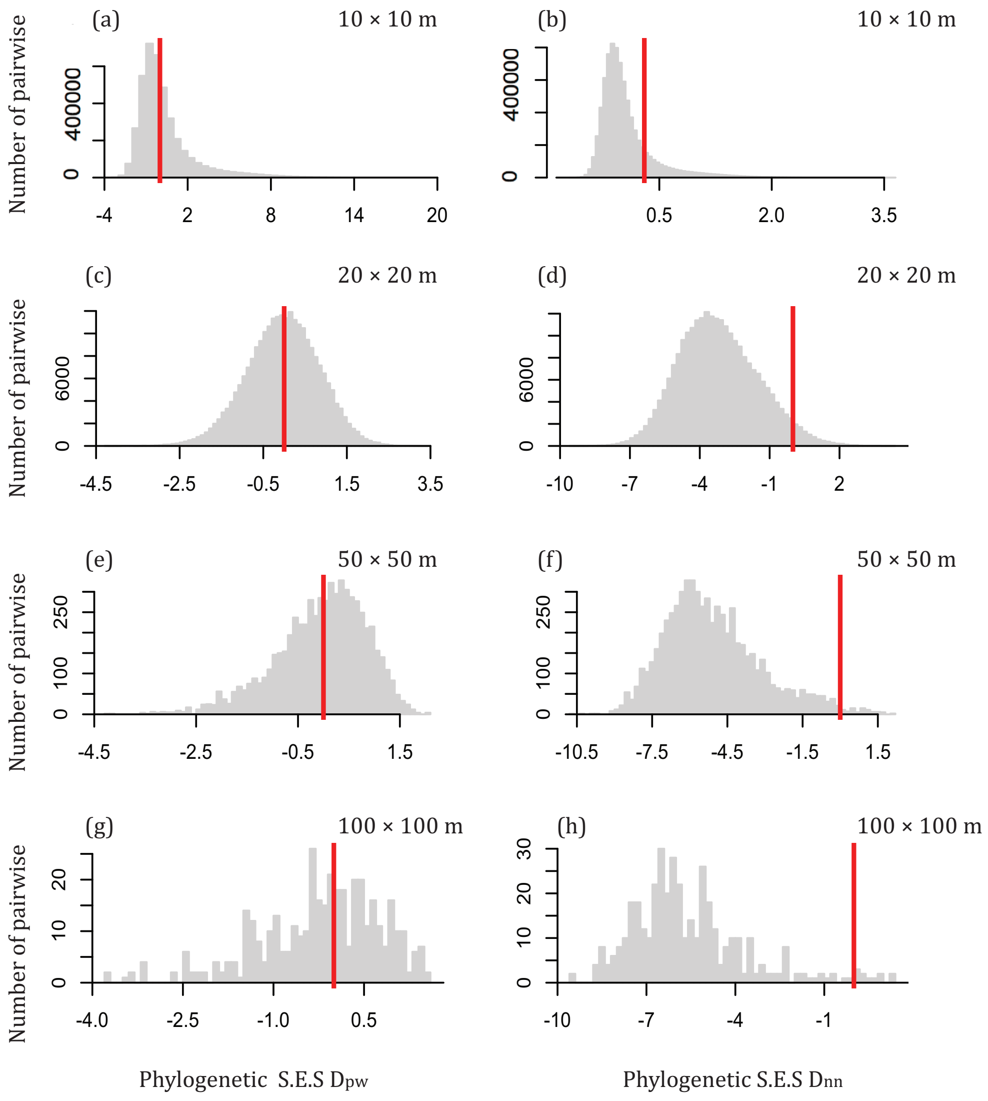

Figure S3 The distribution of standard effective size for non-abundance-weighted mean pairwise phylogenetic distance (*S.E.S.* D_{pw}) and standard effective size for non-abundance-weighted mean nearest taxon phylogenetic distance (*S.E.S.* D_{nn}) across scales. Bars to the left of the red zero line indicates phylogenetic turnover is faster than expected. The proportions of S.E.S values below zero were as follows: (a) 65.16%, (b) 77.35%, (c) 51.28%, (d) 81.76%, (e) 47.80%, (f) 85.81%, (g) 69.75%, (h) 94.56%.

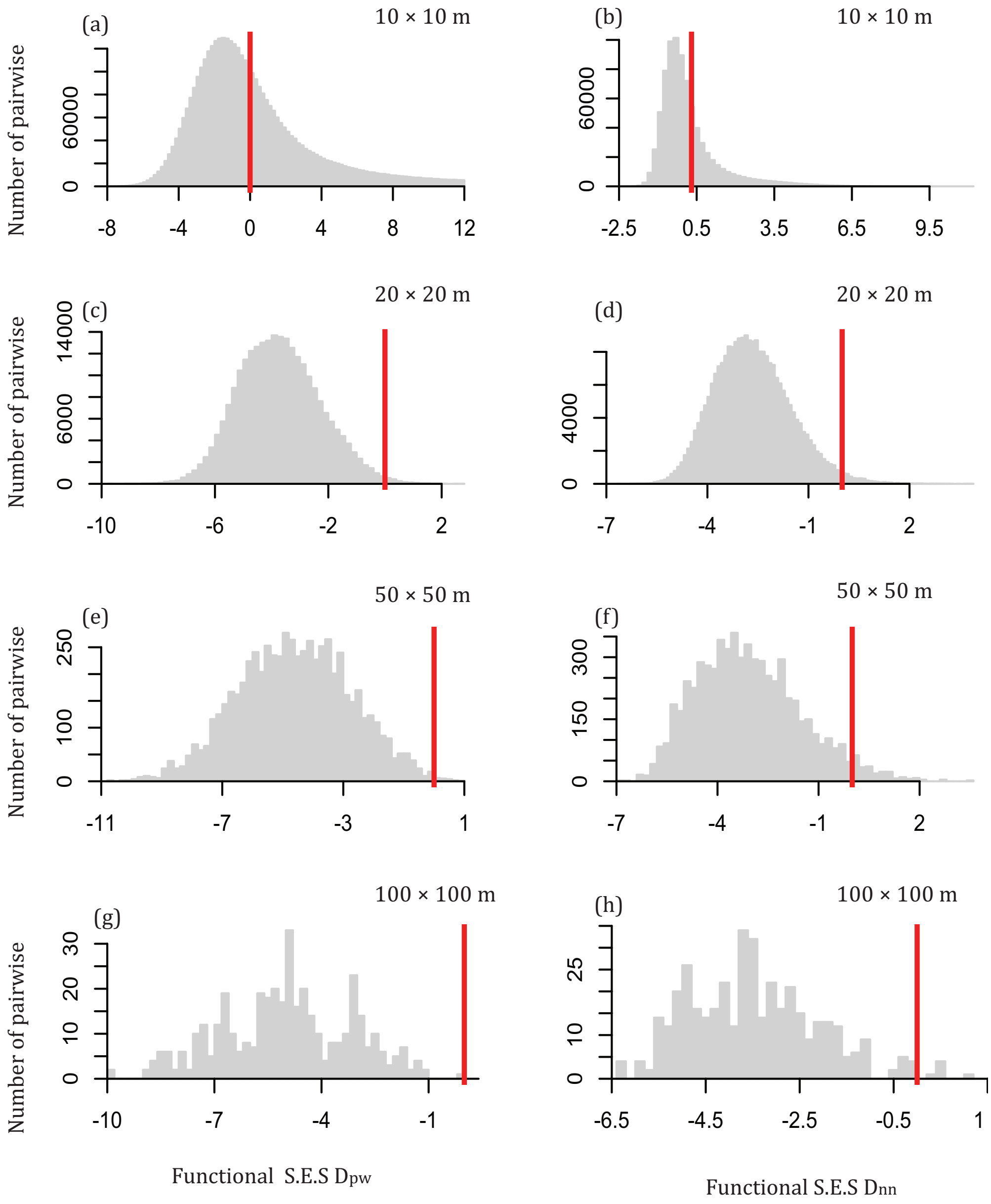

Figure S4 The distribution of standard effective size for non-abundance-weighted mean pairwise functional distance (*S.E.S.* D_{pw}) and standard effective size for non-abundance-weighted mean nearest taxon functional distance (*S.E.S.* D_{nn}) across scales. Bars to the left of the red zero line indicates functional turnover is faster than expected. The proportions of S.E.S values below zero were as follows: (a) 56.35%, (b) 71.26%, (c) 95.6%, (d) 95.48%, (e) 98.02%, (f) 95.91%, (g) 99.6%, (h) 90.25%.
